# Calcium Permeable-AMPA Receptors and Excitotoxicity in Neurological Disorders

**DOI:** 10.3389/fncir.2021.711564

**Published:** 2021-08-17

**Authors:** Changyong Guo, Yao-Ying Ma

**Affiliations:** ^1^Department of Pharmacology and Toxicology, Indiana University School of Medicine, Indianapolis, IN, United States; ^2^Stark Neurosciences Research Institute, Indiana University School of Medicine, Indianapolis, IN, United States

**Keywords:** AMPA receptor, calcium-permeable AMPA receptor, excitotoxicity, neurological disorders, GluA2 subunit

## Abstract

Excitotoxicity is one of the primary mechanisms of cell loss in a variety of diseases of the central and peripheral nervous systems. Other than the previously established signaling pathways of excitotoxicity, which depend on the excessive release of glutamate from axon terminals or over-activation of NMDA receptors (NMDARs), Ca^2+^ influx-triggered excitotoxicity through Ca^2+^-permeable (CP)-AMPA receptors (AMPARs) is detected in multiple disease models. In this review, both acute brain insults (e.g., brain trauma or spinal cord injury, ischemia) and chronic neurological disorders, including Epilepsy/Seizures, Huntington’s disease (HD), Parkinson’s disease (PD), Alzheimer’s disease (AD), amyotrophic lateral sclerosis (ALS), chronic pain, and glaucoma, are discussed regarding the CP-AMPAR-mediated excitotoxicity. Considering the low expression or absence of CP-AMPARs in most cells, specific manipulation of the CP-AMPARs might be a more plausible strategy to delay the onset and progression of pathological alterations with fewer side effects than blocking NMDARs.

## Introduction

Excitotoxicity is one of the primary mechanisms of cell death in a variety of diseases of the central and peripheral nervous systems (CNS and PNS; Plotegher et al., 2021). Elevated Ca^2+^ influx and accumulation inside the cell are the key factors in causing excitotoxicity. Among the diverse sources of Ca^2+^ influx, glutamatergic synaptic transmission-induced increase in cytosolic Ca^2+^ is attributable primarily to: (1) activation of NMDA receptors; (2) activation of voltage-dependent Ca^2+^ channels following membrane depolarization induced by activation of AMPARs, and (3) facilitation of Ca^2+^ release from the intracellular stores by activation of metabotropic glutamate receptors (Pellegrini-Giampietro et al., 1997). The non-NMDA excitatory receptors, including AMPA/Kainate receptors, have been generally considered as Ca^2+^ impermeable based on the fact that the estimated Ca^2+^ permeability of non-NMDARs is about 100-fold lower than that of NMDARs (Mayer and Westbrook, 1987). The impermeability of AMPARs to Ca^2+^ was challenged by the observation that, besides the low Ca^2+^ permeable AMPARs, AMPARs with high permeability exist in a small, unidentified subset of cultured hippocampal neurons (Iino et al., 1990). Since then, the role of CP-AMPARs in physiological and pathological conditions has been studied extensively. Particular emphasis is placed on the role of CP-AMPARs in excitotoxicity, which is an irreversible pathological neural alteration widely detected in neurological disorders (Dong et al., 2009; Selvaraj et al., 2018; Ghirardini et al., 2020; Hu et al., 2020). Although a few recent reviews discussed the potential roles of CP-AMPARs in brain diseases (Kwak and Weiss, 2006; Wright and Vissel, 2012), the present review covers a number of neural mechanisms related not only to RNA editing but also other regulations at the transcriptional and post-translational levels. We first delineate the basic properties of AMPARs and CP-AMPARs, followed by an in-depth discussion on CP-AMPAR-mediated excitotoxicity. In addition to a general overview of mechanisms leading to upregulation of CP-AMPARs, both the effects of acute brain insults (e.g., brain trauma or spinal cord injury, ischemia) and chronic neurological disorders, including epilepsy/seizures, Huntington’s disease (HD), Parkinson’s disease (PD), Alzheimer’s disease (AD), amyotrophic lateral sclerosis (ALS), chronic pain, and glaucoma, are reviewed.

## AMPA Receptors

**Figure 1:**
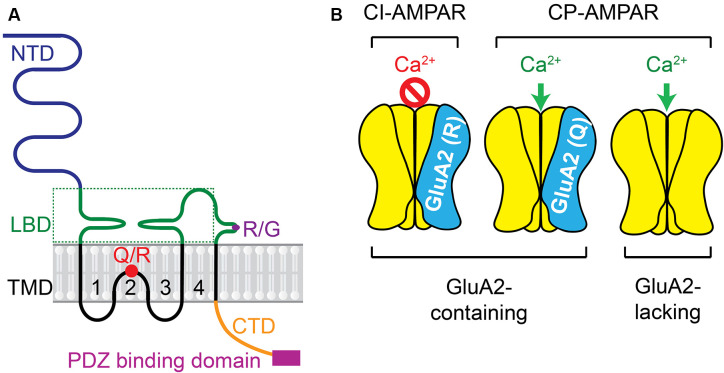
AMPAR subunit and the receptor complex. **(A)** Each subunit has four domains including: (1) the large N-terminal extracellular domain (NTD); (2) the highly conserved extracellular clamshell-like ligand-binding domain (LBD) and (3) transmembrane domains (TMD) 1–4, among which the TMD2 is a re-entry or hairpin loop that forms the pore-lining region and responsible for the channel properties of the receptor, (4) the C-terminal intracellular domain (CTD). Two pre-mRNA editing sites, Q/R and R/G, are presented. More details about Q/R editing are in Figure 3. **(B)** Ca^2+^ permeability of AMPAR complex varies depending on the presence of the GluA2 subunit in the tetrameric complex and the presence of the positively charged R residue in the GluA2-containing AMPARs.

AMPARs are the key subtype of ionotropic glutamate receptors mediating fast synaptic transmission at excitatory synapses in the CNS and PNS. The activity of AMPARs is not only crucial to neuronal development and synaptic plasticity in physiological conditions, but also critical in the induction of neuronal death in neuropathological states. AMPARs are integral transmembrane proteins assembled by GluA1–4 subunits (also named GRIA1–4 or GluR1–4) as a tetrameric complex. Each subunit has four blocks of domains (Greger et al., 2017; Purkey and Dell’Acqua, 2020; Cull-Candy and Farrant, 2021) including, first, a large N-terminal extracellular domain (NTD) which drives the multimerization and is responsible for inducing dendritic spine morphogenesis; second, a highly conserved extracellular clamshell-like ligand-binding domain (LBD) which, together with NTD, comprise ~85% of receptor mass and protrude ~130 Angstroms into the synaptic cleft; third, transmembrane domains (TMD) 1–4, among which the TMD2 is a re-entry or hairpin loop that forms the pore-lining region and responsible for the channel properties of the receptor; fourth, the C-terminal intracellular domain (CTD) which, together with other domains, determines the receptor assembly and trafficking (Figure 1A). AMPARs subunit composition affects affinity, kinetics, ionic permeability, and channel conductance, leading to receptors with *sui generis* characteristics.

## CP-AMPARs

The Ca^2+^ conductance of AMPARs varies depending on whether the GluA2 subunit is present in the tetrameric complex (Figure 1B). Usually, GluA2-lacking AMPARs are Ca^2+^−permeable (CP). CP-AMPARs are mostly atypical AMPARs, which are different from GluA2-containing Ca^2+^ impermeable (CI)-AMPARs that are typically found in the mature brain (Man, 2011). The presence of GluA2 subunit in AMPARs prevents the influx of cations such as Ca^2+^ and Zn^2+^. Within the TMD2, the GluA2 subunit usually contains the positively charged arginine (R) residue at the Q/R site, which is not encoded at the genomic level but arises owing to RNA editing (Liu and Zukin, 2007). A codon for the neutral glutamine (Q) residue in GluA2 pre-messenger RNA in the nucleus is catalyzed predominantly by the adenosine deaminase acting on RNA type 2 (ADAR2) enzyme (Sommer et al., 1991; Burnashev et al., 1992; Higuchi et al., 1993). This Q/R editing is done very efficiently in nearly 100% GluA2 subunits in mammalian neurons (Kawahara et al., 2003, 2004b, 2005), whereas the equivalent position of other AMPAR subunits (GluA1, GluA3, and GluA4) is usually preserved as Q in its unedited form (Wright and Vissel, 2012).

CP-AMPARs could function to amplify biological signals. The presence of the positively charged R residue in the GluA2-containing AMPARs renders the channel impermeable to Ca^2+^, slows the channel kinetics, decreases the channel conductance, enhances the amplitude of synaptic events and paired-pulse facilitation of synaptic currents, increases neuronal excitability, and regulates the trafficking and synaptic anchoring of AMPARs (Geiger et al., 1995; Swanson et al., 1997; Carter and Regehr, 2002; Bats et al., 2013). Together with the low level of CP-AMPARs in most of the principal neurons in the adult brain, even a modest alteration in expression levels of CP-AMPARs is expected to have profound implications for synaptic transmission and circuit remodeling (Liu and Zukin, 2007).

## CP-AMPARs and Excitotoxicity

Ca^2+^ influx through CP-AMPARs is thought to play a critical role in synaptogenesis and formation of neuronal circuitry during early development (McDonald and Johnston, 1990; Stubblefield and Benke, 2010). In the adult brain, the CP-AMPARs are not typically present in most cells of the CNS and PNS, but their temporary increase may contribute to activity-dependent synaptic plasticity and the related behavioral phenotypes (Di et al., 2019; Manz et al., 2020; Alonso-Caraballo et al., 2021; Yu et al., 2021).

Excitotoxicity, defined as pathological over-activation of excitatory neurotransmission, is thought to contribute to synaptic and neuronal degeneration (Beal, 1992; Liu Y. et al., 2019; Olajide et al., 2021). One of the misconceptions about neuronal excitotoxicity is that elevated glutamate levels must be detected (Wang et al., 2014). Excess release of glutamate from axon terminals is a hallmark of acute brain injuries with fast and severe neural tissue damage (Choi and Rothman, 1990; Tejeda-Bayron et al., 2021). However, the accumulated glutamate is not necessarily a characteristic of a slowly progressing excitotoxicity, which is more often the case in neurodegenerative diseases. Another misconception is that NMDARs are the primary or exclusive source of the Ca^2+^ influx.

NMDAR activation leads to excitotoxicity at the cellular and circuit levels both *in vivo* and *in vitro*. While there is a general consensus that deranged Ca^2+^ homeostasis is the main cause of synapse and cell loss (Bezprozvanny and Hayden, 2004), the exact source of excessive intracellular Ca^2+^ flux remains obscure. After years of intensive investigation on NMDA receptors (NMDARs) in synaptic/neuronal excitotoxicity, there are several strong rationales to switch our attention from NMDARs to AMPARs, particularly the CP-AMPARs. First, NMDAR activation is essential for normal neuronal function. Administration of NMDAR blockers as anti-excitotoxicity neuroprotective agents can block virtually all NMDAR activity, leading to unacceptable clinical side effects. Second, Memantine, a partial antagonist of NMDARs and well-tolerated in patients was approved in both Europe and the USA for the treatment of Alzheimer’s disease (AD)-associated dementia. However, clinically memantine showed limited beneficial effects. Third, in contrast to NMDARs, which are usually inactive at resting membrane potential due to the channel blockade by Mg^2+^, CP-AMPARs are not blocked by extracellular cations and allow Ca^2+^ entry at any level of receptor activation. In fact, CP-AMPARs increase significantly in diseased states and they are permeable to Zn^2+^, which can trigger mitochondrial dysfunction and cell death (Sensi et al., 1999; Jia et al., 2002; Redman et al., 2009; Huang et al., 2011). Fourth, CP-AMPARs increase in neurodegenerative disease models (Whitehead et al., 2017), but NMDARs decrease, although in a few cases they may also increase, during neural degeneration (Malinow, 2012; Avila et al., 2017; Foster et al., 2017; Wang and Reddy, 2017; Liu J. et al., 2019), strongly suggesting more persistent effects of excitotoxicity from CP-AMPARs. Fifth, blockade of CP-AMPARs by CP- AMPAR antagonists (e.g., 1-Naphthylacetyl spermine trihydrochloride, Naspm) is neuroprotective in brain insults (Noh et al., 2005). Sixth, CP-AMPARs may be considered as an early marker indicating the onset of pathological progression in neurological diseases. CP-AMPARs are expressed transiently at high levels in synaptic membranes at the early developmental stage, followed by a significant drop in mature neurons (Wenthold et al., 1996; Rozov et al., 2012). Growing evidence demonstrates that CP-AMPARs have a low probability of being detected in mature brains unless there are associated pathological events such as epilepsy (Malkin et al., 2016; Sun et al., 2018), ischemia (Noh et al., 2005; Kwak and Weiss, 2006), traumatic brain injury (Spaethling et al., 2008) and drug abuse (Ma et al., 2014, 2016, etc.). Recently, accumulating evidence supports the role of CP-AMPARs in neurological diseases (Whitehead et al., 2017).

## Mechanisms of Upregulation of CP-AMPARs

Substantial evidence shows that not only AMPAR subunit composition, but also the Q/R RNA editing in the GluA2 subunit are regulated developmentally, and also by synaptic activity or neuropathological alterations (Seeburg and Hartner, 2003; Kawahara et al., 2005; Kwak and Weiss, 2006). In general, the CP-AMPARs may be upregulated by transcription of AMPAR subunits, the pre-mRNA editing before translation, and post-translational modification including AMPAR assembly and trafficking.

### Dysregulation of AMPAR Subunit Expression

**Figure 2:**
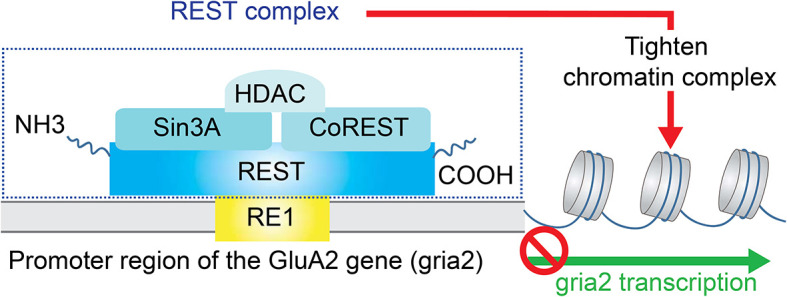
Repression of GluA2 transcription. The gene silencing transcription factor neuronal repressor element-1 (RE1) silencing transcription factor (REST) represses the transcription of the AMPAR subunit GluA2. Specifically, REST binds the cis-acting RE1 within the promoter region of the GluA2 gene. Together with the co-repressors Sin3A and coREST which recruit histone deacetylase (HDAC), the REST complex silences GluA2 gene transcription by deacetylation of the core histone protein and tightening of the core chromatin complex.

Deficiency or relatively low expression of GluA2 subunits increases the probability of AMPAR assembly with no GluA2 subunits. These GluA2-lacking AMPARs are the primary type of CP-AMPARs in the CNS and PNS. One of the potential signaling pathways in mediating down-regulation of GluA2 subunit expression is chromatin remodeling by transcriptional repression (Figure 2). The gene silencing transcription factor neuronal repressor element-1 (RE1) silencing transcription factor (REST) represses the transcription of a subset of neural genes (Schoenherr and Anderson, 1995; Calderone et al., 2003; Noh et al., 2012). This repressive DNA regulatory element prevents the expression of neuronal genes important to synaptic plasticity including the AMPAR subunit GluA2 (Calderone et al., 2003; Noh et al., 2012; Butler-Ryan and Wood, 2021). Consisting of nine non-canonical zinc finger motifs, REST binds the cis-acting RE1 within the promoter region of the GluA2 gene (Chong et al., 1995; Schoenherr et al., 1996). Together with the co-repressors Sin3A and coREST which recruit histone deacetylase (HDAC), the REST complex silences GluA2 gene transcription by deacetylation of the core histone protein and tightening of the core chromatin complex.

Selectively reducing the expression of GluA2 leads to an increase of CP-AMPARs in multiple disease models (see more details below). Interestingly, enhanced expression of synaptic CP-AMPARs in cultured hippocampal neurons from GluA2-null mice shows no increase in the risk of cell death (Iihara et al., 2001). GluA2-deficient mice are mostly viable with no significant neuronal death, although they show upregulated CP-AMPARs, impairments in motor coordination, and open field behavior (Jia et al., 1996). Double knock-out of both GluA2 and GluA3 was not fatal in rodents and show no evidence of neuronal death (Meng et al., 2003). These results suggest that the presence of a GluA2 subunit *per se* is not essential for neuronal survival but that a reduction in pre-existing GluA2 expression may prime neuronal death (Kawahara and Kwak, 2005).

If the protein level of GluA1 is modified too, the relatively higher ratio of GluA1:GluA2, but not the absolute protein level of GluA1 or GluA2, is directly associated with increased CP-AMPARs (Kondo et al., 2000). Increase of GluA1:GluA2 ratio may serve as a molecular switch in the formation of GluA2 lacking CP-AMPARs (Pellegrini-Giampietro et al., 1997; Di et al., 2019). Furthermore, similar to GluA1 subunits, the upregulation of GluA4 subunits also contributes to the expression of CP-AMPARs and synaptic strength during hyperalgesia (Taylor et al., 2019).

### Incomplete Q/R Editing of GluA2 Subunits

**Figure 3:**
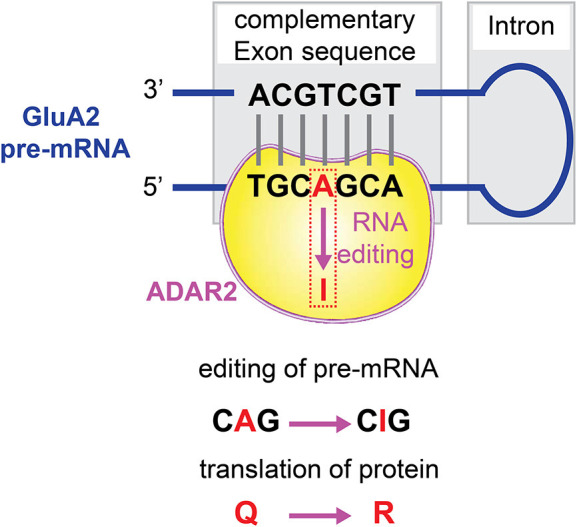
Q/R editing of GluA2 pre-mRNA. The RNA processing machinery ADAR2 binds to the double strands of GluA2 pre-mRNA, and catalyzes A-to-I conversion at the GluA2 glutamine/arginine (Q/R) site.

Q/R editing at the Q/R site of GluA2 subunit pre-mRNA is catalyzed predominantly by the RNA processing machinery ADAR2 (Melcher et al., 1996; Higuchi et al., 2000; Wang et al., 2000; Figure 3). The expression level of ADAR2 is proportionally correlated with the editing efficiency at the GluA2 Q/R site in the CNS (Kawahara et al., 2003, 2004b). Neurons sensitive to degenerative insults to the brain expressed ADAR2 enzyme at a lower level, insufficient to allow GluA2 RNA Q/R site editing (Peng et al., 2006; Wang et al., 2014). The stable ADAR2 gene silencing by delivering siRNA inhibits GluA2 Q/R site editing, could mimic the neurodegeneration-associated cellular effects. On the other hand, direct introduction of the Q/R site edited GluA2 gene, and constitutive activation of CREB, induced the expression of ADAR2 and prevented ADAR2-associated degeneration. Thus, the mRNA editing machinery can be regulated to modify the channel properties of AMPARs.

### Dysregulation of AMPAR Assembly and Trafficking

**Figure 4:**
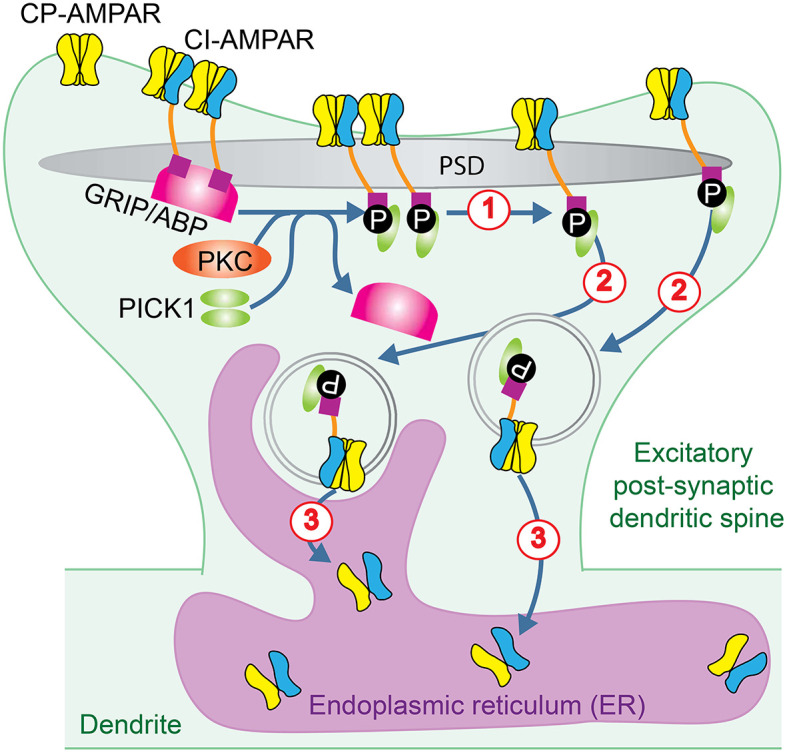
AMPAR trafficking regulated by PKC. PKC phosphorylation of GluA2 subunit in GluA2-containing CI-AMPARs disrupts the association of GRIP/ABP from GluA2 CTD and facilitates its association with PICK1. This rearrangement of CTD binding proteins results in: (1) disruption of AMPAR clusters at the synaptic membrane; (2) internalization of synaptic GluA2 subunits, and (3) **restriction** of GluA2 subunits to the intracellular pool in the ER.

The vast majority of the tetrameric AMPAR complex consists of GluA1/2 and GluA2/3 heteromers. Excitatory synaptic transmission primarily depends on the individual AMPAR channel property, which is directly determined by the GluA2 subunit, and the available number of AMPARs. AMPAR assembly could be regulated by the availability of GluA2 subunits, relative to the available non-GluA2 subunits. Concentrated GluA2 subunits in the endoplasmic reticulum (ER) form an intracellular pool outside of the synapses as a storage of this critical subunit, serving for at least two functions including, first, to ensure the availability of GluA2 for AMPA receptor assembly, and second, to facilitate incorporation of GluA2 subunits during AMPAR assembly (Greger et al., 2002; Pick and Ziff, 2018). The GluA2 subunit assembly and trafficking are regulated exquisitely by various proteins. For example, postsynaptic density protein 95/disc large/zona occludens-1 (PDZ) domain of anchoring proteins, such as Glutamate receptor-interacting protein (GRIP) 1 and GRIP2 / AMPAR binding protein (ABP), protein interacting with C kinase 1 (PICK1) bind to the CTD of GluA2 subunits (Shi et al., 2001; Barry and Ziff, 2002). The relationship between GRIP/ABP and PICK1 is well orchestrated by activation of PKC (Matsuda et al., 2000). PKC phosphorylation of GluA2 serine residue S880 in its PDZ-binding motif disrupts the association of GRIP/ABP from GluA2 CTD, followed by increased association of PICK1 to GluA2 CTD (Carroll et al., 2001). It was hypothesized that this rearrangement of CTD binding proteins results in, first, disruption of AMPAR clusters at the synaptic membrane, second, internalization of synaptic GluA2 subunits, and third, restraint of GluA2 subunits to the intracellular pool in the ER (Figure 4). Thus, activation of GRIP/ABP and PICK1 anchoring protein results in the internalization of nonCP-AMPARs and more CP-AMPARs available at the synaptic membrane. Another example is that the transmembrane AMPA receptor regulatory proteins (TARPs) play different roles in delivering CP-AMPARs, relative to nonCP-AMPARs (Cull-Candy and Farrant, 2021). The absence of TARP γ-2 increases the surface expression of CP-AMPARs (Bats et al., 2012). Extrasynaptic CP-AMPARs are associated with TARP γ-7, which can further enhance the synaptic expression of CP-AMPARs (Studniarczyk et al., 2013). Thus, increased cytosolic assembly and synaptic distribution of CP-AMPARs may enhance Ca^2+^ influx, triggering excitotoxicity.

## Critical Involvement of CP-AMPARs in Neurological Disorders

Upregulation of CP-AMPARs, together with their biological amplifying effects, triggers the Ca^2+^ influx excitotoxic signaling pathway, which may result in mitochondrial injury, ER-stress, activation of apoptotic cascades, and cell death (Araujo et al., 2004). Consequently, the cellular integrity is more likely to be broken down, followed by alterations in the ubiquitin-proteasome system. Pathological responses of the altered ubiquitin-proteasome system lead to further neuronal cell death (Caldeira et al., 2014).

In this review, involvement of CP-AMPARs in both acute brain insults (e.g., brain trauma or spinal cord injury, ischemia) and chronic neurological disorders, including epilepsy/seizures, Huntington’s disease, Parkinson’s disease, Alzheimer’s disease, amyotrophic lateral sclerosis, chronic pain, and glaucoma, are reviewed. Excitotoxicity is recognized as one of the primary pathological alterations in these neurological disorders. Thus, the potential participation of CP-AMPARs in these disorders is discussed.

### Brain Trauma or Spinal Cord Injury

Injury to the CNS and PNS is characterized by a series of biochemical events spreading from the initial injury site and leading to the death of cells in neighboring, initially undamaged tissue. Cell death after traumatic brain injury (TBI) occurs in various cell types and in multiple brain regions. This post-traumatic cell loss is the major cause of TBI-associated neurological deficits and mortality (Stoica and Faden, 2010; Ng and Lee, 2019; Akamatsu and Hanafy, 2020; Barrett et al., 2020). Glutamatergic synaptic transmission is implicated in TBI (Shohami and Biegon, 2014; O’Neil et al., 2018; Sloley et al., 2021) as exemplified in the following studies.

Cerebellar Purkinje neurons’ vulnerability to brain trauma is attributed to CP-AMPAR-mediated Ca^2+^ overload, which initiates biochemical cascades that ultimately cause progressive cell death (Bell et al., 2007). A rapid emergence of CP-AMPARs occurs 15 min after mild mechanical injury, combined with a modest AMPAR stimulation. Inhibition of PKC-dependent GluA2 endocytosis or the CP-AMPAR antagonist Naspm eliminates the enhancement of CP-AMPARs. Further immunocytochemical analysis shows downregulation of GluA2 signals co-localized with the pre-synaptic marker synaptophysin. In another set of experiments, 4 h after TBI, cortical neurons show a gradual expression of CP-AMPARs, which is mediated by CaMKII-dependent phosphorylation (Spaethling et al., 2008). Additionally, Naspm blocks the injury-induced loss of cortical neurons.

Peripheral nerve injury increases GluA2 internalization, and surface expression of CP-AMPARs is increased in the dorsal horn neurons (Chen et al., 2016). Blocking NMDARs, calpain or calcineurin, but not PKC, abolishes CP-AMPAR-mediated synaptic transmission in nerve-injured rats. Direct inhibition of CP-AMPARs by the selective antagonist IEM-1460 attenuates the nerve injury-induced pain hypersensitivity (Chen et al., 2013).

The involvement of CP-AMPARs in multiple injury systems by targeting different neuronal populations indicates that CP-AMPAR-mediated cell loss is not unique to one model system or one specific region, but is broadly implicated in the neuronal responses to traumatic injury of the CNS and PNS (Goforth et al., 2011; Spaethling et al., 2012; Korgaonkar et al., 2020). Blockade of CP-AMPAR function, for example through prevention of GluA2 endocytosis, may be critical in the development of therapies for CNS or PNS injury, and potentially avoid ensuing secondary damage (Bell et al., 2007).

### Ischemia

In equally affected brain regions during transient, but severe global ischemia, all neurons experience hypo-oxygenation and glucose deprivation, but only selected neurons such as pyramidal neurons in CA1, degenerate and die, which is not detected until 48–72 h after circulation is restored. AMPAR antagonists such as NBQX, appear to be more effective than NMDAR antagonists in preventing cell death. This delayed damage is attributable to a reduction in expression of mRNA encoding the AMPAR subunit GluA2 (Pellegrini-Giampietro et al., 1992), indicating enhancement of CP-AMPARs and increased vulnerability of these neurons. More recently, other groups also found an increase in the prevalence of Ca^2+^-permeable GluA2-lacking AMPARs in hippocampal CA1 neurons after ischemic damage (Quintana et al., 2015; Koszegi et al., 2017; Mazzocchetti et al., 2020).

Global ischemia also triggers REST mRNA and protein expression (Calderone et al., 2003). As we discussed above, REST suppresses promoter activity and transcription of the GluA2 gene. Acute knockdown of the REST gene by antisense manipulation prevented GluA2 suppression and rescued post-ischemic neurons from ischemia-induced cell death. Thus, inhibition of GluA2 expression after ischemia insults by REST-dependent transcription silencing, which consequently increases CP-AMPARs, is one of the important mechanisms of neuronal death.

On the other hand, the increase of non-GluA2 subunits (e.g., GluA4) detected in *in vitro* ischemia model might be associated with decreased expression of GluA2 (Pellegrini-Giampietro et al., 1997). It is worth noting that loss of GluA2 could be brain region- and/or cell type-specific. For example, it seems a preferential loss of GluA2 immunoreactivity is not detected for selective neurodegeneration in amacrine and ganglion cells after retinal ischemia (Dijk and Kamphuis, 2004).

The forebrain ischemia in adult rats selectively reduces expression of ADAR2 enzyme and, hence, disrupts pre-mRNA Q/R site editing of GluA2 subunits in vulnerable neurons of the rat hippocampus. Recovery of GluA2 Q/R site editing by expression of exogenous ADAR2b gene or a constitutively active CREB, VP16-CREB, which induces expression of endogenous ADAR2, protects vulnerable neurons from forebrain ischemic insult (Peng et al., 2006). Generation of a stable ADAR2 gene silencing by delivering small interfering RNA (siRNA) inhibits GluA2 Q/R site editing, leading to the degeneration of ischemia-insensitive neurons. Direct introduction of the Q/R site edited GluA2 gene, GluA2 (R607), rescues ADAR2 degeneration. Thus, ADAR2-dependent GluA2 Q/R site editing determines the vulnerability of neurons in the rat hippocampus to forebrain ischemia, which is also confirmed by analysis of a new mouse line with reduced GluA2 Q/R site RNA editing (Konen et al., 2020).

Brain ischemia promotes internalization of GluA2-containing AMPARs from the post-synaptic membrane *via* clathrin-dependent endocytosis and facilitates the synaptic insertion of GluA2-lacking AMPARs *via* soluble N-ethylmaleimide-sensitive factor attachment protein receptor-dependent exocytosis (Liu et al., 2006). This ischemia-induced switch in AMPAR subunit composition requires PKC activation, dissociation of GluA2 from AMPAR-binding protein (ABP), and association with protein interacting with C kinase-1 (PICK1; Figure 4). The inserted GluA2-lacking CP-AMPARs may be relevant to ischemia-induced synaptic remodeling and neuronal death.

### Epilepsy/Seizures

Epilepsy is a chronic CNS disorder that causes recurrent seizures or periods of unusual behavior, sensations, and sometimes loss of awareness. Synaptically connected excitatory glutamatergic neurons play critical roles in the abnormally synchronous discharges when epileptic seizures occur (Rogawski, 2013). Recent studies identified that CP-AMPARs may contribute to neural dysfunction and the related excitotoxicity in epilepsy (Shao et al., 2018; Konen et al., 2020; Postnikova et al., 2020).

One of the mechanisms of increased CP-AMPARs in epilepsy is the silencing of GluA2 transcription. As the RE1 repressor protein, REST is rapidly induced in the hippocampus after seizures (Palm et al., 1998; Spencer et al., 2006; McClelland et al., 2014; Patterson et al., 2017; Navarrete-Modesto et al., 2019). REST-mediated recruitment of HDAC to the GluA2 gene inhibits the transcription process. Huang et al. found that HDAC inhibitor traichostatin A prevented seizure-induced deacetylation of GluA2-associated histones, leading to a decrease of GluA2 protein levels (Huang et al., 2002).

Another pathway to upregulate CP-AMPARs is to target the pre-mRNA and interrupt the Q/R editing of GluA2 subunits before translation. GluA2 Q/R insufficient editing in principal neurons expressing CP-AMPARs induces seizures in mice at the age of 3 weeks (Brusa et al., 1995) or 8–10 weeks (Konen et al., 2020). In the latter study, heterozygous mice with a point mutation in the pre-mRNA editing complementary sequence of the GluA2 gene display ~ 20% reduction in GluA2 RNA editing at the Q/R site, increases of CP-AMPAR expression, and decreases of dendritic spine density in the CA1 pyramidal neurons. Behaviorally, these mice exhibit NMDAR-independent seizures, which are blocked by CP-AMPAR antagonist IEM-1460. However, the human data about the Q/R editing of GluA2 subunits have been reported inconsistently. For example, Q/R unedited GluA2 subunits were detected in surgically excised hippocampus from young patients (up to 10-year old), but those with a long history of epilepsy or control samples showed complete Q/R editing in GluA2 (Grigorenko et al., 1998). The autopsy tissue from epileptic patients showed complete Q/R editing of GluA2 subunits in the hippocampus and temporal cortex, similar to that in the normal controls (Kortenbruck et al., 2001). We conclude that the involvement of CP-AMPARs in epilepsy depends on the stage of disease progression and the age of subjects as well.

### Huntington’s Disease

Huntington’s disease (HD) is an inherited, ultimately fatal neurodegenerative condition for which there is currently no cure. It is caused by an anomalous expansion of CAG repeats coding for glutamine (The Huntington’s Disease Collaborative Research Group, 1993). HD is characterized by motor alterations, e.g., chorea and dystonia, which have been related to dysfunction in the dorsolateral striatum. To date, there is no definitive answer as to why the striatum is the most vulnerable in HD. A role for glutamate receptor-mediated excitotoxicity was suggested more than 25 years ago (DiFiglia, 1990). Since then, the main focus of research has been on the glutamate NMDA receptor subtype (Raymond et al., 2011). However, exclusive focus on NMDA receptors has hampered examination of other potential excitotoxicity mechanisms as well as the development of new therapies for HD.

In HD, a decrease in GluA2 subunits is observed in the putamen from post-mortem brain tissue, suggesting alterations in AMPAR-mediated synaptic transmission in the basal ganglia. CP-AMPARs with a high conductance to Ca^2+^ could consequently play a predominant role in the motor symptoms of HD (Fourie et al., 2014) and in neuronal damage (Mandal et al., 2011; Rocher et al., 2016). In BACHD mice, for example, the ratio of *GluA1/GluA2* mRNA is higher than in control animals, suggesting the increase of CP-AMPARs (Rocher et al., 2016). Further, in human HD patients, it was demonstrated that the Q/R unedited form of *GluA2* is higher than in controls (Akbarian et al., 1995). Striatum from HD patients has ~5% of all GluA2 mRNA deficient in Q/R editing, which is significantly higher than the Q/R non-editing level (i.e., 0.5%) in striatum from controls with no neurodegenerative or psychiatric disorders (Akbarian et al., 1995). Thus, the aforementioned evidence indicates CP-AMPARs are enhanced in HD. More studies need to be done to identify the neural mechanisms of CP-AMPAR enhancement in HD and to evaluate whether the upregulated CP-AMPARs facilitate neuronal loss in HD.

### Parkinson’s Disease

Parkinson’s disease (PD) is an age-related disorder caused by the degeneration and loss of dopaminergic neurons within the substantia nigra pars compacta (Ambrosi et al., 2014). Excitotoxicity is detected in dopaminergic pathways from the substantia nigra pars compacta to the striatum in PD animal models. Most of the mechanistic studies of PD-associated excitotoxicity have focused on increased glutamate release from presynaptic terminals and persistent activation of postsynaptic NMDARs. Similar to the field of HD research, the Ca^2+^ source is proposed to be limited to NMDAR-dependent pathway. The involvement of CP-AMPARs in the Ca^2+^ influx-triggered excitotoxic consequences on the dopamine neurons in the basal ganglia has rarely been explored to date. However, and interestingly, a couple of PD-associated disease models have identified the involvement of CP-AMPARs in pathological alterations. First, CP-AMPARs are involved in the expression of L-DOPA-induced dyskinesia in PD (Kobylecki et al., 2010). Second, the CP-AMPAR-induced loss of dopamine neurons in the midbrain causes PD-related depression (Zhang et al., 2019). A potential mechanism could be that increased expression of CaMKIIβ in the lateral habenula (LHb) was upregulated in the PD models where the mesocortical DA pathway displayed degeneration. Blockade of CP-AMPARs in the LHb prevented DA neuron death, increased DA release in the prefrontal cortex, and produced antidepressant effects. Thus, a role for CP-AMPARs in PD is beginning to be gleaned and more studies are warranted.

### Alzheimer’s Disease

Alzheimer’s disease (AD), a devastating neurodegenerative disease with no known cure, is the most common cause of dementia (Hill et al., 2002), but its pathological determinants are still debated. It is hypothesized that the AD brain pathology starts with massive synaptic dysfunction, followed by neuronal degeneration and death (Andrade-Moraes et al., 2013). The prefrontal cortex from AD patients has ~1% of all GluA2 mRNA showing no Q/R editing, which is significantly higher than the Q/R non-editing level (i.e., < 0.1%) in controls with no neurodegenerative or psychiatric disorders (Akbarian et al., 1995). Postsynaptic density-rich fractions from AD patients’ hippocampi showed a significant increase of GluA1 subunits relative to healthy controls (Marcello et al., 2012). Whitcomb et al. (2015) showed that oligomerized Aβ induces a rapid enhancement of synaptic CP-AMPAR insertion in hippocampal slices. CP-AMPARs may be considered as an early marker indicating the onset of pathological progression in AD-associated neurodegeneration and cell loss (Whitehead et al., 2017). Thus, similar to PD, an important role of CP-AMPARs can be expected in the early stages of AD. The early interruption of CP-AMPAR-mediated neurodegeneration could delay the pathological progression in AD patients.

### Amyotrophic Lateral Sclerosis

As a typical progressive neurodegenerative disease, amyotrophic lateral sclerosis (ALS) affects motor neurons of the spinal cord, brain stem, and the pyramidal cells of the motor cortex. AMPAR-mediated excitotoxicity, attributed to Ca^2+^ influx through CP-AMPARs, is implicated in the selective motor neuron loss and thus the development of ALS (Rothstein et al., 1990, 1993; Carriedo et al., 1995; Ikonomidou et al., 1996; Bar-Peled et al., 1999; Tateno et al., 2004; Hideyama et al., 2010; Yamashita and Kwak, 2014, 2019; Selvaraj et al., 2018). Different from other CNS areas and most neuronal subsets, the spinal motor neurons express a relatively low level of GluA2 subunits and possess a relatively high level of CP-AMPARs. This unique characteristic of normal spinal motor neurons is a major determinant of the selective vulnerability of these neurons to excitotoxicity (Van Damme et al., 2002). At least two possible neural mechanisms causing excitotoxicity in spinal motor neurons have been identified (Kawahara and Kwak, 2005). First, a further reduction in the expression of GluA2 subunits, or increased expression of nonGluA2 subunits, leads to an increased ratio between nonGluA2:GluA2 subunits in the spinal motor neurons and results in a relative overabundance of CP-AMPARs, thereby increasing the risk for excitotoxicity. Overexpression of edited GluA2 subunits in spinal motor neurons delays the ALS onset and its mortality (Tateno et al., 2004). For example, induced pluripotent stem cell (iPSC)-derived motor neurons from ALS patients carrying the C9ORF72 mutation exhibit increased expression of GluA1 subunits, which leads to increased CP-AMPAR expression and results in the enhanced selective vulnerability of motor neurons to excitotoxicity. This vulnerability is abolished by CRISPR/Cas9-mediated correction of the C9ORF72 mutation. Interestingly, AMPAR expression is selectively dysregulated in the spinal cord, but not cortical, post-mortem tissue from patients carrying C9ORF72 mutation (Selvaraj et al., 2018). Second, a reduction of GluA2 Q/R editing in the motor neurons leads to a relatively low abundance of edited GluA2 subunit in the AMPAR complex (Kawahara et al., 2004a), thus the Ca^2+^ permeability is relatively high. For example, expression levels of ADAR2 in motor neurons of sporadic ALS are reduced, which results in insufficient Q/R RNA editing in GluA2 subunits. TAR DNA-binding protein (TARDBP) 43, denoted TDP-43 (trans-active response DNA binding protein, 43 kDa), shuttles between the nucleus and cytoplasm, thereby playing a role in transcriptional regulation and alternative splicing (Ou et al., 1995; Wang et al., 2004; Lukavsky et al., 2013). TDP-43 pathology is the most reliable hallmark of motor neuron pathology of ALS, in which TDP-43 is abnormally insoluble, mislocalized, hyperphosphorylated, and fragmented in motor neurons (Arai et al., 2006; Neumann et al., 2006; Kwong et al., 2007; Chen-Plotkin et al., 2010). Notably, TDP-43 pathology down-regulates ADAR2, leading to a failure in Q/R editing of GluA2 pre-mRNA (Yamashita and Kwak, 2019). This results in significant losses of motor neurons in the majority of sporadic ALS patients (Aizawa et al., 2010; Hideyama et al., 2012).

From the above studies on different neurodegenerative diseases, a common thread becomes apparent. The emergence or increase in the density of CP-AMPARs on the cell membrane appears to trigger an excitotoxic cascade that only later implicates NMDARs and voltage-gated Ca^2+^ channels. This allows early therapeutic intervention to delay or even prevent cells’ demise. However, the importance of CP-AMPARs is not limited to these common neurodegenerative disorders, as explained below.

### Chronic Pain

Chronic pain is persistent pain lasting longer than 6 months, which can continue even after the injury or illness that caused it has healed or disappeared. Intrathecal pretreatment with AMPAR antagonists reduces pain perception (Sorkin et al., 2001; Nozaki-Taguchi and Yaksh, 2002). The activation of spinal AMPAR might contribute to sensitization under inflammation-induced persistent pain conditions. Although total GluA1 protein level in the spinal cord was not affected, peripheral inflammatory insults increased the membrane expression of GluA1 and decreased cytosolic levels of GluA1 subunits (Galan et al., 2004; Choi et al., 2010). Further studies on the inflammation pain model demonstrated that the GluA2 subunits were redistributed with a shift from the membrane to the cytosolic compartment with no changes in its total protein level (Park et al., 2008). These data support the hypothesis of replacement of membrane GluA2-containing AMPARs by GluA2-lacking AMPARs, indicating an increase of synaptic CP-AMPARs in mediating chronic pain. Such upregulation can last up to 21 days after the induction of inflammatory pain (Taylor et al., 2019). Spinal CP-AMPARs appear to drive activity-dependent changes in synaptic processing of nociceptive inputs. Spinal CP-AMPAR inhibition reverses opioid receptor agonist naltrexone-induced reinstatement of pain. Therefore, CP-AMPAR inhibitors have been considered as promising agents for the treatment of chronic pain.

The activation of phosphatidylinositol 3-kinase is necessary for the membrane recruitment of GluA1 subunit in dorsal horn neurons (Pezet et al., 2008). The internalization of GluA2 subunits is initiated by GluA2 phosphorylation at Serine (S) 880, S831, and S845 by PKC and subsequent disruption of GluA2 binding to its synaptic anchoring proteins such as ABP/GRIP, stargazin, and PICK1 (Osten et al., 2000; Fang et al., 2003; Nagy et al., 2004; Park et al., 2009). NMDAR-triggered PKC activation is involved in the GluA2-internalization (Park et al., 2009). Besides PKC, other kinases, such as PKA and CaMKII, have also been reported in phosphorylating GluA1 subunits in chronic pain models (Barria et al., 1997; Choi et al., 2010; Peng et al., 2011).

Using patch-clamp recordings combined with Ca^2+^ imaging and cobalt staining, the nociception-induced enhancement of CP-AMPARs in dorsal horn neurons was identified with an enriched distribution in the extra-synaptic membranes (Kopach et al., 2011).

CP-AMPARs in excitatory and inhibitory neurons could be modified differently (Kopach et al., 2015; Chen et al., 2016; Taylor et al., 2019). For example, chronic constriction injury produced a loss of synaptic CP-AMPARs on inhibitory neurons but not on excitatory neurons (Chen et al., 2016), indicating that the effect of nerve injury acting on synapses of inhibitory neurons might disrupt homeostasis between excitation and inhibition of neural network. Thus, chronic pain-induced upregulation of CP-AMPARs alters brain function in diverse ways.

### Glaucoma

Glaucoma is the second leading cause of blindness overall, and the leading cause of blindness in the African American and Hispanic communities (Quigley and Broman, 2006; Stein et al., 2021). No dramatic enhancement of synaptic glutamate neurotransmitters occurs through the chronic and gradual course of glaucoma. However, intraocular pressure selectively reduces expression of the ADAR2 enzyme and disrupts GluA2 Q/R site editing in a mouse model of glaucoma (Wang et al., 2014). Restoring the ADAR-based RNA processing machinery may be a novel target for the prevention and treatment of glaucoma, which was verified by genomic editing of the GluA2 Q/R site in retinal ganglion cells (Sladek and Nawy, 2020). Furthermore, elevated TNF-alpha as a mechanism regulating AMPAR expression was also reported (Almasieh et al., 2012; Cueva Vargas et al., 2015). Specifically, NMDAR-dependent activation of NF-kB in Müller cells led to the production of endogenous glia-derived TNFa which, in turn, rendered RGCs highly sensitive to excitotoxicity by increasing their surface levels of CP-AMPARs (Lebrun-Julien et al., 2009). Thus, effective prevention of CP-AMPAR-mediated excitotoxicity can be achieved by targeting both mRNA editing and the surface expression of AMPARs.

## Conclusions

CP-AMPAR-mediated excitotoxicity represents a common mechanism in multiple disease model systems. CP-AMPAR-associated pathological alterations could induce neural excitotoxicity in different brain regions, neural circuits, cellular types, as well as various intracellular signaling pathways, all of which may correspondingly lead to some unique manifestations of neurological diseases. For example, CP-AMPAR increase in the amygdala was related to anxiety (Yi et al., 2017); in the lateral habenula it was linked to depression (Zhang et al., 2019); in hippocampus and cortices, it was implicated in seizures (Lippman-Bell et al., 2016), ischemia (Dias et al., 2013), AD (Whitehead et al., 2017), and schizophrenia (Umino et al., 2018); in the dorsal horn neurons, it was involved in chronic pain (Kopach et al., 2011), whereas, in the dorsal and ventral striatum, the CP-AMPAR increase was associated with HD (Mandal et al., 2011) and drug addiction (Ma et al., 2014), respectively. Further exploration of CP-AMPARs in CNS and PNS diseases will not only expand our knowledge of each pathological process but also improve our understanding of how the nervous system regulates diverse signaling pathways in a biologically economic manner by regulating a shared synaptic event, i.e., CP-AMPAR-mediated synaptic transmission. Considering the low density or even absence of CP-AMPARs in most areas of the CNS and PNS, as opposed to NMDARs which are more widely expressed, manipulation of the CP-AMPARs might reverse early and relevant pathological alterations and demonstrate clinical benefits with much less undesired side effects.

## Author Contributions

Manuscript preparation: CG and Y-YM. All authors contributed to the article and approved the submitted version.

## Conflict of Interest

The authors declare that the research was conducted in the absence of any commercial or financial relationships that could be construed as a potential conflict of interest.

## Publisher’s Note

All claims expressed in this article are solely those of the authors and do not necessarily represent those of their affiliated organizations, or those of the publisher, the editors and the reviewers. Any product that may be evaluated in this article, or claim that may be made by its manufacturer, is not guaranteed or endorsed by the publisher.
